# Construction of a genome instability-derived lncRNA-based risk scoring system for the prognosis of hepatocellular carcinoma

**DOI:** 10.18632/aging.203698

**Published:** 2021-11-18

**Authors:** Dan-Ping Huang, Mian-Mian Liao, Jing-Jing Tong, Wei-Qu Yuan, De-Ti Peng, Jian-Ping Lai, Yi-Hao Zeng, Yi-Jun Qiu, Guang-Dong Tong

**Affiliations:** 1 Department of Hepatology, Shenzhen Traditional Chinese Medicine Hospital, The Fourth Clinical Medical College of Guangzhou University of Chinese Medicine, Shenzhen 518033, Guangdong Province, China; 2 College of Basic Medicine, Guangzhou University of Chinese Medicine, Guangzhou 510403, Guangdong, China; 3 The Affiliated Chencun Hospital of Shunde Hospital, Southern Medical University, Shunde 528300, Guangdong Province, China; 4 Department of Acupuncture, Shenzhen Traditional Chinese Medicine Hospital, The Fourth Clinical Medical College of Guangzhou University of Chinese Medicine, Shenzhen 518033, Guangdong Province, China; 5 The First Affiliated Hospital of Guangzhou University of Chinese Medicine, Guangzhou 510403, Guangdong Province, China

**Keywords:** long non-coding RNAs, genomic instability, prognosis prediction, hepatocellular carcinoma, The Cancer Genome Atlas

## Abstract

Emerging evidence revealed the critical roles of long non-coding RNAs (lncRNAs) in maintaining genomic instability. However, genome instability-associated lncRNAs (GILncRNAs) and their performance in clinical prognostic significance in hepatocellular carcinoma (HCC) are rarely reported. Our study constructed a computational framework integrating somatic mutation information and lncRNA expression profiles of HCC genome and we identified 88 GILncRNAs of HCC. Function enrichment analysis revealed that GILncRNAs were involved in various metabolism processes and genome instability of cancer. A genome instability-derived lncRNA-based gene signature (GILncSig) was constructed using training set data. The performance of GILncSig for outcome prediction was validated in testing set and The Cancer Genome Atlas (TCGA) set. The multivariate cox regression analysis and stratification analysis demonstrated GILncSig could serve as an independent prognostic factor for the overall survival of HCC patients. The time-dependent Receiver Operating Characteristic (ROC) curve illustrated GILncSig outperformed two recently published lncRNA signatures for overall survival prediction. The combination of GILncSig and tumor protein p53 (TP53) mutation status exhibited better prognostic performance in survival evaluation compared to TP53 mutation status alone. AC145343.1 was further validated to be a risk factor for HCC *in vitro among GILncSig*. Overall, our study provided a novel approach for identification of genome instability-associated lncRNAs and established an independent risk score system for outcome prediction of HCC patients, which provided a new insight for exploring in-depth mechanism and potential therapy strategy.

## INTRODUCTION

Liver cancer is the second leading cause of tumor-related death and disability-adjusted living-years causing more than 700,000 deaths each year [1]. There are about 840,000 people suffering from liver cancer, but the death toll is as high as 780,000 every year worldwide, most of which occur in developing countries according to the global cancer statistics in 2018 [2]. Although sensitivity and specificity of early diagnosis of liver cancer have improved to a certain extent, the 5-year survival rate is still only about 17%. Hepatocellular carcinoma (HCC) as the most important type of liver cancer accounting for 85% to 90% is critical for liver cancer research. Liver resection and liver transplantation are the main treatments for HCC, but only a few patients are eligible for surgery and approximately 70% of patients undergoing surgery will relapse within 5 years after surgery [3]. In addition, the symptoms of HCC commonly appear late leading to most HCC patients being diagnosed with advanced cancer accompanied by intrahepatic or distant metastasis with poor effect of surgical treatment and the prognosis of HCC is still very poor with a 5-year survival rate of less than 20% [4, 5]. To improve clinical outcomes for patients, serum biomarkers of HCC were being continuously developed. An abnormal increased level of plasma AFP in adults is considered to be a hallmark of pathological conditions of HCC [6]. AFP is overexpressed in more than 70% of clinical HCC patients. Serum AFP level has been considered the ‘gold standard’ biomarker for clinical liver cancer diagnosis over the last few decades [7]. A protein induced by vitamin K absence or antagonist-II (PIVKA-II) is also useful for the diagnosis of early HCC and has been used as a predictive marker of microvascular invasion [8]. In addition, 1 specific type of AFP—AFP-L3—binds to a lectin and displays serum levels that are in consistent with levels of AFP in human sera [9]. AFP-L3 can be used to differentiate an increase in AFP due to HCC or benign liver disease [10–12]. However, these biomarkers at the protein level are still limited by low sensitivity and specificity [13]. Genome instability, an increase in the tendency to acquire genomic changes ranging from base pair mutations to chromosomal aberrations, contributes to somatic cell heterogeneity and genetic diversity as a material for natural and artificial selection, while it also contributes to the progression of genetic related diseases including cancer [14, 15]. Genome instability is the basic feature of tumor cells and the core sign of tumorigenesis, and the evolution from early atypical hyperplasia to malignant and metastatic tumors is often accompanied by increasing genomic instability [16]. Moreover, genomic instability is closely related to tumor progression and affects prognosis and survival [17]. The causes of genome instability are very complicated which may be associated with replication dysfunction, DNA repair failure, abnormal transcription, various metabolism process and post-transcriptional regulation.

It is clear that long non-coding RNA (lncRNA) is becoming a potential regulator and quantitative measurement of genome instability [18, 19]. LncRNA mostly transcribed by RNA polymerase II without protein-coding function is defined as an RNA transcript with more than 200 nucleotides located in the nucleus or cytoplasm. LncRNA participates in cell cycle, differentiation, cell migration, invasion, proliferation and apoptosis, and functions as a cell microstructure original and small RNA precursor [20, 21]. Transcription and dysfunction of lncRNA is closely involved in tumorigenesis, including HCC [22]. Moreover, lncRNA with time and tissue specificity shows different expression levels in tumors and healthy states and different tumor stages, which suggests lncRNA has great potential to be a new prognostic biomarker [23]. For example, a controlled study involving 80 HCC patients and 50 healthy subjects finds that the expression of cancer susceptibility candidate 9 in HCC patients significantly increases with area under the curve (AUC) at 0.933 [24]. Study shows the volume of liver cancer tumors reduces by 82% in animal models of liver cancer when H19 (the first reported lncRNA) is knocked out, which fully confirms the tumorigenic effect of H19 [25]. Short-term recurrence after liver resection or liver transplantation in patients with HCC is related to the increasing expression of HOTAIR and the decreased expression of HOTAIR leads to the apoptosis of liver cancer cell lines [26]. The above researches show that lncRNAs are closely related to the progression of HCC, but the mechanism of regulating HCC is still elusive. Evidence shows that lncRNA participates in gene expression at the transcription and post-transcriptional levels, thus involves in regulating genomic instability [27]. However, genome instability-associated lncRNAs (GILncRNAs) and their clinical prognostic significance in HCC are rarely reported.

In this study, we constructed a computational framework integrating somatic mutation profiles and lncRNA expression profiles of HCC to recognize GILncSig for HCC, and confirmed the performance of GILncSig on HCC. We further validated the biology function of the most important lncRNA of the GILncSig-AC145343.1 with Cell Counting Kit-8 (CCK-8), colony formation, transwell and wound healing assay. Our study revealed a novel approach for identification of genome instability-associated lncRNAs and established an independent signature for outcome prediction of HCC.

## RESULTS

### Identification of GILncRNAs in HCC patients

**Figure 1:**
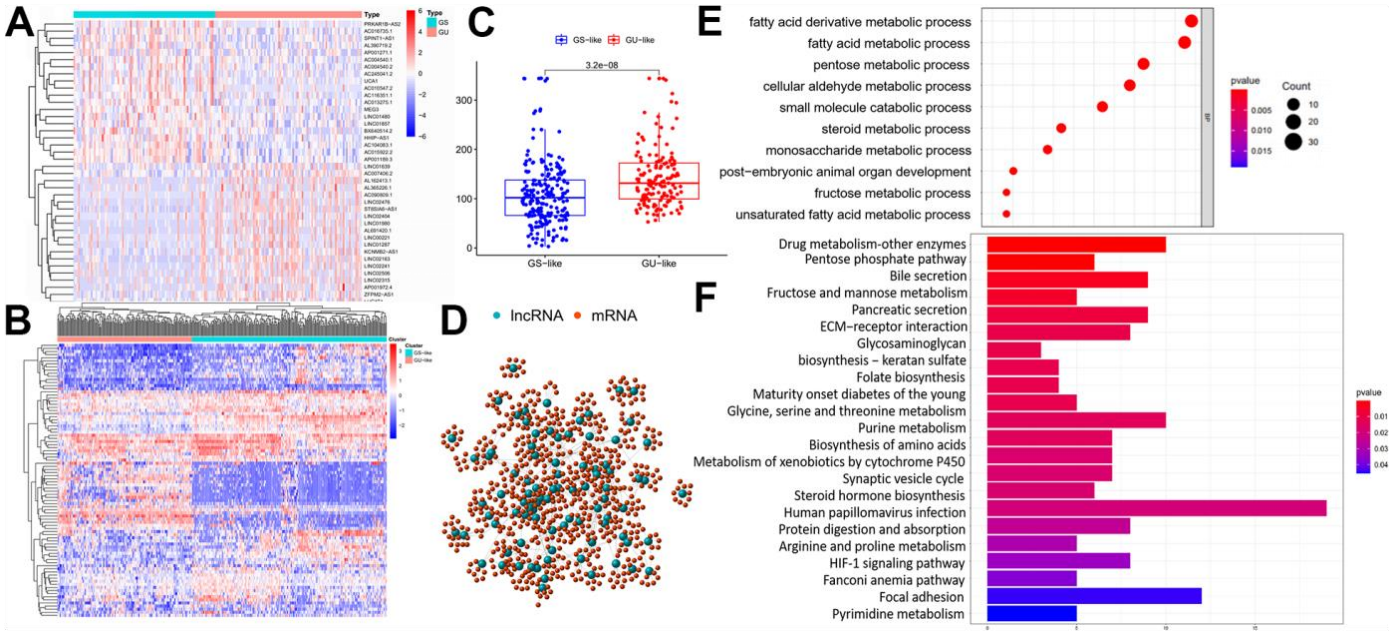
**Identification and functional annotations of genomic instability-related lncRNAs.** (**A**) GU-like group and GS-like group identification according to the top 25% samples and the last 25% ones in the ranking list of cumulative somatic mutations. The left blue cluster is GS-like group, and the right red cluster is GU-like group. (**B**) Unsupervised clustering of 364 HCC patients based on the expression pattern of 88 candidate genomic instability-related lncRNAs. The left orange cluster is GU-like group, and the right blue cluster is GS-like group. (**C**) Boxplots of somatic mutations in the GU-like group and GS-like group. Somatic cumulative mutations in the GU-like group are significantly higher than those in the GS-like group. (**D**) Coexpression network of genomic instability-related lncRNAs and mRNAs. The red circles represent mRNAs, and the blue circles represent lncRNAs. (**E**, **F**) Functional enrichment analysis of GO and KEGG for mRNAs co-expressed lncRNAs.

The cumulative somatic mutations for each sample were first computed and ranked in the decreasing order. The top 25% samples and the last 25% ones in the ranking list were defined as genomic unstable (GU)-like group and genomic stable (GS)-like group, respectively (Figure 1A). Each group was consisted of 91 samples. Next, significant expressed lncRNAs were identified by comparing the lncRNA expression profiles between GU-like group and GS-like group. 88 lncRNAs were obtained based on the criteria of |fold change| > 1.5 and FDR adjusted *P*<0.05 and served as GILncRNAs. Of these, 32 lncRNAs were upregulated and 56 were downregulated in GU-like group (Supplementary Data 2). Then, we performed unsupervised hierarchical clustering analysis of all 364 samples using the filtered expression profile of differently expressed GILncRNAs. All samples were hence re-grouped based on the cluster result (Figure 1B). The group with higher cumulative somatic mutations was defined as GU-like group, and the other group was named as GS-like group. As shown in Figure 1B, the somatic mutation pattern was significantly different between the two groups. The median value of somatic cumulative mutations was 156.7 in the GU-like group while 124.0 in the GS-like group (*P*<0.001) (Figure 1C).

To validate the potential function of identified 88 lncRNAs and uncover their association with genomic instability, Gene Ontology (GO) terms and Kyoto Encyclopedia of Genes and Genomes (KEGG) analysis were carried out using clusterProfiler software in R-version 3.5.2. Firstly, protein coding genes (PCGs) closely related to the expression of lncRNAs were screened out using Pearson Correlation Coefficients. The top 10 PCGs most correlated with each lncRNAs were retained for lncRNAs–mRNA co-expression network construction. In the co-expression network, the nodes represented lncRNAs and mRNAs, and the lines represented the relationships between lncRNAs and mRNAs (Figure 1D). Go analysis of the PCGs in the network indicated that genomic instability might impact various metabolism process including fatty acid / fatty acid derivative metabolic process, pentose metabolic process, cellular aldehyde metabolic process, small molecule catabolic process and so on (Figure 1E). In terms of KEGG pathway analysis, PCGs in the network were found to enriched in a variety of metabolism pathway, HIF-1 signal pathway, extracellular matrix-receptor interaction, biosynthesis of amino acids and so on (Figure 1F). Enrichment results further reveal genomic instability-related lncRNAs were involved in various biological process of cancer. Expression of lncRNA could break the regulatory balance between lncRNA and PCGs, subsequently interfere with a variety of metabolism pathway, leading to gene damage repair process and exacerbation of genomic instability.

### Construction of GILncSig for outcome prediction in the training set

To explore the role of GILncRNAs in the prognosis of HCC patients, 343 samples downloaded from TCGA were randomly divided into 2 groups named the training set (n=172) and testing set (n=171) respectively. As shown in Supplementary Table 1, there was no significant difference observed in the common clinical features characteristic between two groups (*P* > 0.05, Chis-square test). Then training set were used to establish GILncSig of HCC patients. We performed univariate Cox proportional hazard regression analysis to investigate the association of GILncRNAs and overall survival (OS) of HCC patients in the training set and found 9 GILncRNAs were closely related to the prognosis of HCC patients (*P*<0.05; Supplementary Table 2). Furthermore, multivariate Cox regression analysis was performed to evaluate the independent prognostic value of 9 GILncRNAs. 3 of 9 candidate lncRNAs including AC145343.1, AC004862.1 and ZFPM2-AS1 were obtained with prognostic significance in multivariate Cox analysis (*P*<0.05) (Table 1). Finally, GILncSig was established to predict outcome of HCC patients in the training set according to the equation mentioned above. The GILncSig was constructed as follow: GILncSig score = (0.3804 × expression level of AC145343.1) + (0.1253 × expression level of ZFPM2-AS1) + (−0.2344 × expression level of AC004862.1). A positive/negative regression coefficient demonstrated a positive/negative association between risk score and the expression level of lncRNA. Higher GILncSig score means higher risk of poor prognosis. Of the GILncSig, AC145343.1 and ZFPM2-AS1 tended to be risky factors while AC004862.1 was more likely to be a protective factor for the survival of HCC patients.

**Table 1:** Multivariate Cox regression analysis.

**Gene symbol**	**Coefficient**	**HR**	**95% CI**	**P-value**
AC145343.1	0.380	1.463	1.134-1.888	0.003
AC004862.1	-0.234	0.791	0.657-0.952	0.013
ZFPM2-AS1	0.125	1.133	1.076-1.193	1.57E-06

The GILncSig of each sample in the training set were calculated and then these patients were equally divided into high risk group and low risk group according to computed risk score with a decreasing order. Next we investigated the survival time of patients in the two groups using Kaplan–Meier analysis. The result showed that longer survival of patients in the low-risk group compared that in the high-risk.

**Figure 2:**
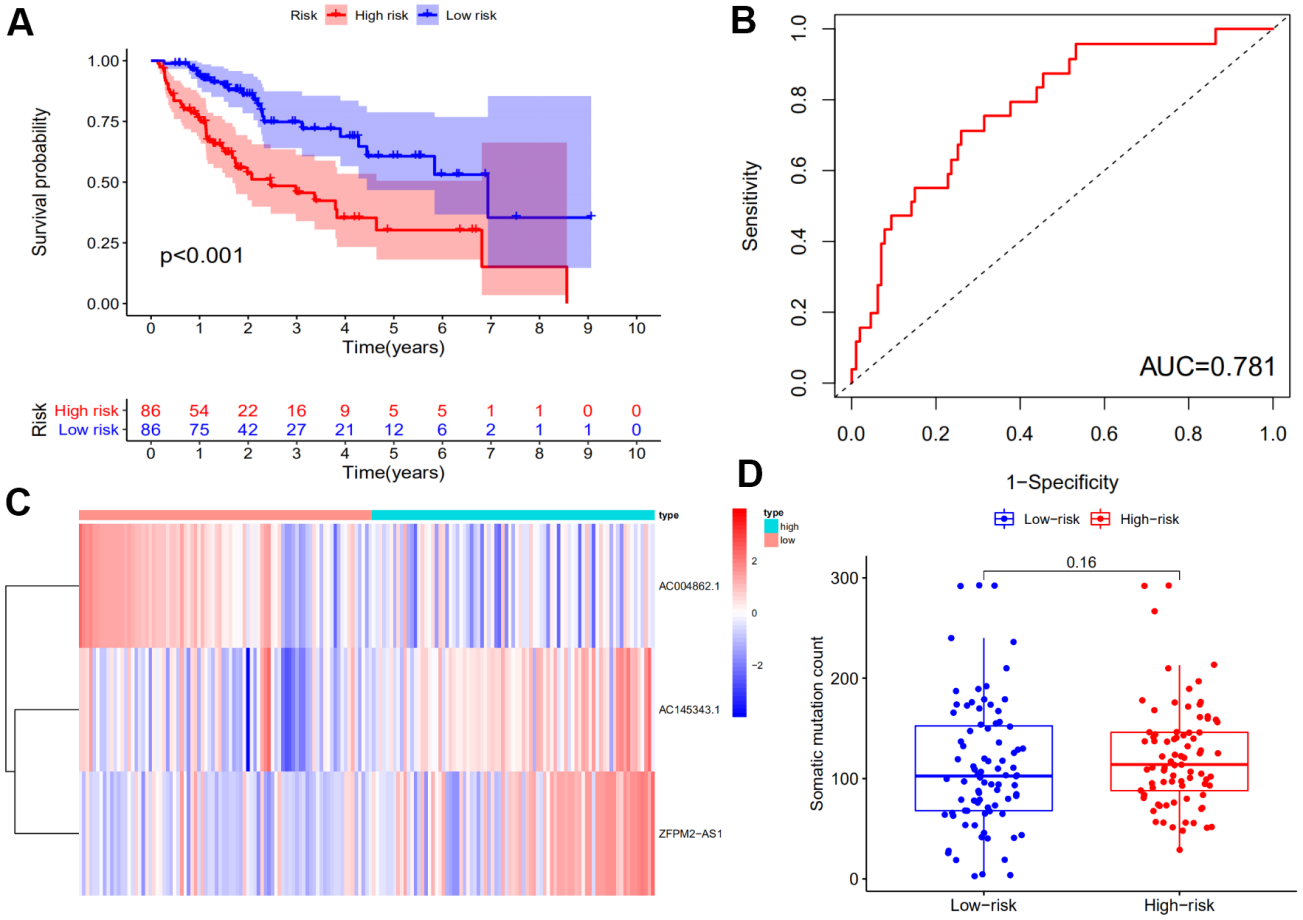
**Identification of the genomic instability-derived lncRNA signature (GILncSig) using the training set.** (**A**) Kaplan–Meier analysis of overall survival of patients with low or high risk according to the GILncSig score in the training set. Statistical analysis was performed using the log-rank test and univariate Cox analysis. (**B**) Time-dependent ROC curves analysis of the GILncSig. (**C**) LncRNA expression patterns with increasing GILncSig score. (**D**) Somatic mutations count in the high- and low-risk groups for the training set patients. The red represents the high-risk group, and the blue represents the low-risk group.

Group (*P*<0.001, log rank test; Figure 2A). The time-dependent ROC curve was illustrated in Figure 2B and demonstrated an AUC of 0.781 for the GILncSig. We also plotted the expression levels of lncRNAs in the GILncSig and the count of somatic mutations with the increasing score in patients of the training set. As shown in Figure 2C, the risk lncRNA AC145343.1 and ZFPM2-AS1 showed up-regulated expression while the protective lncRNA AC004862.1 showed opposing expression pattern in the samples with high risk scores. Comparison analysis showed the number of somatic mutation between two groups has no significance (*P* = 0.16) but the average count of somatic mutation in patients of high-risk group was higher than that of low-risk group (Figure 2D).

### Independent examination of GILncSig for HCC patients

**Figure 3:**
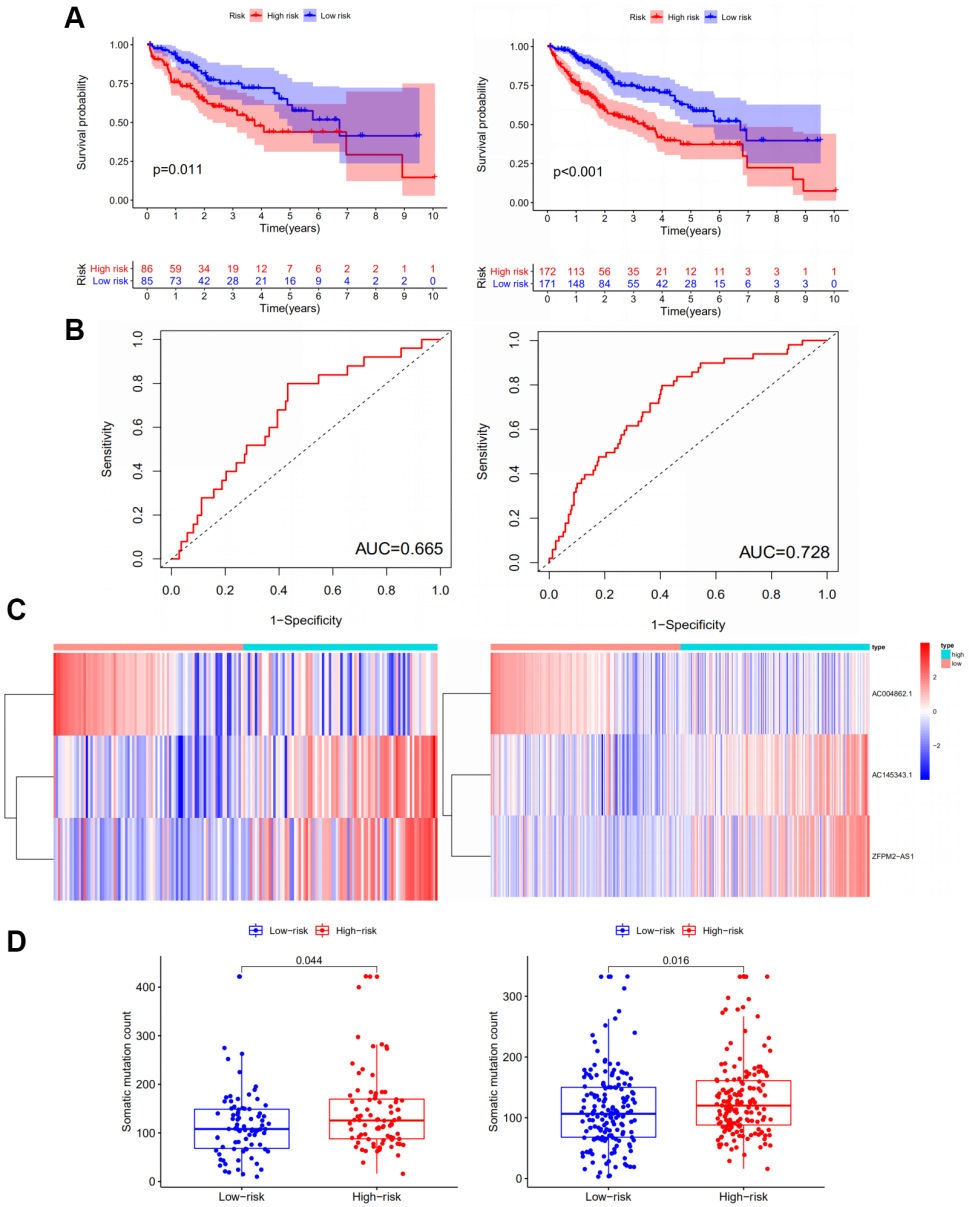
**Performance examination of the GILncSig in the testing set and TCGA set.** (**A**) Kaplan–Meier analysis of overall survival of patients with low or high risk according to the GILncSig score in the testing set (Left panel) and TCGA set (Right panel). Statistical analysis was performed using the log-rank test and univariate Cox analysis. (**B**) Time-dependent ROC curves analysis of the GILncSig in the testing set (Left panel) and TCGA set (Right panel). (**C**) LncRNA expression patterns with increasing GILncSig score in the testing set (Left panel) and TCGA set (Right panel). (**D**) Somatic mutations count in the high- and low-risk groups for the patients in the testing set (Left panel) and TCGA set (Right panel). The red represents the high-risk group, and the blue represents the low-risk group.

The RNA-seq data of testing group including 171 HCC samples was analyzed to assess the stability, validity and prediction ability of the GILncSig. Patients in the testing group were also evenly separated into the high-risk group and low-risk group based on their GILncSig score. Kaplan–Meier survival analysis showed that patients in the high-risk group had a shorter survival time compared to those in the low-risk group (*P*<0.05) (Figure 3A; Left panel). The time-dependent ROC curves analysis of the GILncSig in the testing set yielded an AUC of 0.665 (Figure 3B; Left panel). Then we sorted the samples in the testing group according to GILncSig score with the increasing order to further demonstrate the alteration of the expression levels of the GILncSig (Figure 3C; Left panel). Concurring with the expectation, the risk lncRNAs AC145343.1 and ZFPM2-AS1 were more highly expressed and the protective lncRNA AC004862.1 showed lower expression pattern in the patients of testing set with higher risk scores. The number of the distribution of somatic mutation in patients with high scores was significantly higher than in patients with low scores (*P*<0.05, Figure 3D; Left panel).

Similar results have been obtained in the whole TCGA samples in terms of the prognostic value of the GILncSig. Kaplan–Meier plot analysis demonstrated that patients in the low-risk group had a more favorable survival time compared to those in the high-risk group (*P*<0.001) (Figure 3A; Right panel). As for ROC curve, the TCGA set yielded an AUC of 0.728 (Figure 3B; Right panel). The expression patterns of lncRNAs AC145343.1, ZFPM2-AS1 and AC004862.1 in TCGA set were consistent with that in the training and testing group (Figure 3C; Right panel). The high-risk group also showed increased somatic mutation counts compared to the low-risk group (*P*<0.05) (Figure 3D; Right panel).

### Independence of the GILncSig from common clinical variables

**Figure 4:**
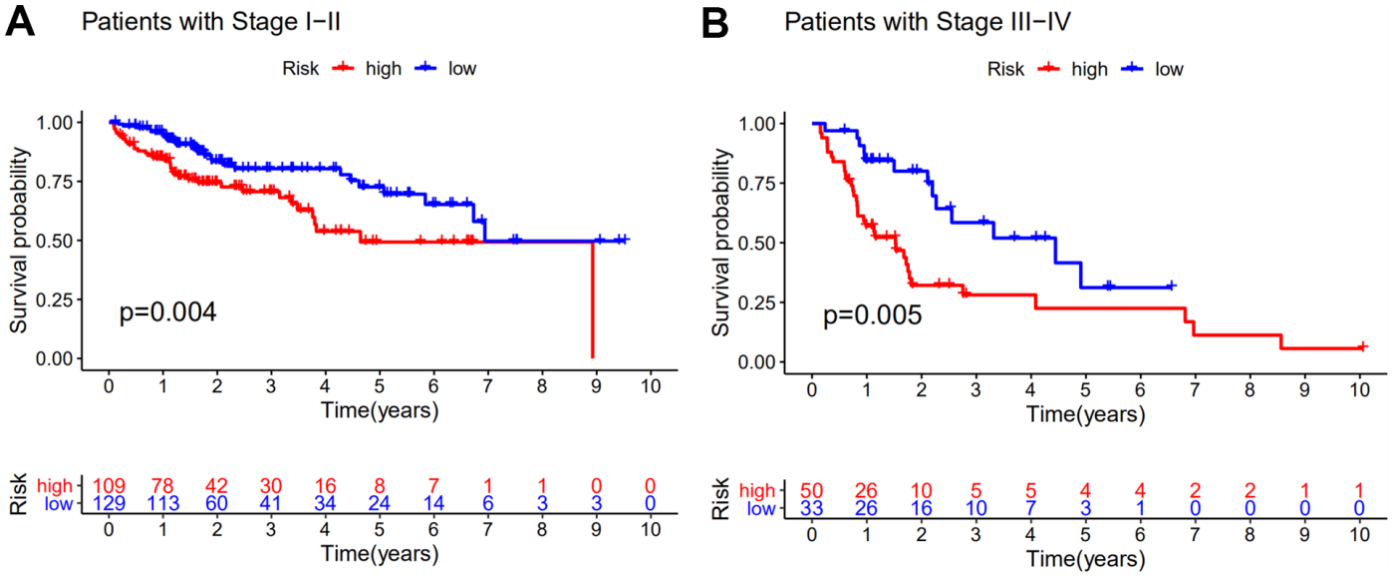
**Stratification analyses by stage.** Kaplan–Meier curve analysis of overall survival of patients in high- and low-risk groups for early-stage patients (**A**) and late-stage patients (**B**). Statistical analysis was performed using the log-rank test and univariate Cox analysis.

To access the prognostic value of other clinical factors, we first conducted univariate Cox regression analyses on age, gender, tumor grade, tumor stage and GILncSig of each set. The result showed that only tumor stage and GILncSig were closely related to the survival time of HCC patients in each set (Table 2). Multivariate Cox regression was further analyzed to explore the independence performance of the GILncSig. Tumor stage and GILncSig also exhibited significant difference in each group based on the multivariate analysis result (Table 2). Then we continued to examine whether the prognosis performance of GILncSig was independent of the tumor stage. We excluded the patients with unknown stage and stratified remaining samples into early-stage group (stage I-II, n = 238) and late-stage group (stage III-IV, n = 83) for stratification analysis. Samples in each group were further separated into high- risk and low-risk group. A significant difference in survival outcome between high-risk and low-risk group was observed in both early-stage group and a late-stage group (*P*<0.01) (Figure 4). It was clear that the GILncSig could serve as an independent prognostic factor for the overall survival of HCC patients.

**Table 2:** Univariate and multivariate Cox regression analysis of the GILncSig and overall survival in different patient sets.

**Variables**		**Univariable model**			**Multivariable model**		
		**HR**	**95% CI**	**P-value**	**HR**	**95% CI**	**P-value**
Training set (n =182)							
GILncSig	High/Low	1.129	1.076	8.37E-07	1.153	1.096-1.213	2.96E-08
Age		1.004	0.984	1.025			
Gender		0.721	0.419	1.241			
Grade		1.300	0.932	0.122			
Stage		1.811	1.353	6.51E-05	1.931	1.428-2.611	1.90E-05
Testing set (n =182)							
GILncSig	High/Low	1.023	0.922-1.136	6.64E-03	1.102	1.042-1.145	2.23E-03
Age		1.007	0.986-1.029	0.497			
Gender		0.792	0.451-1.391	0.417			
Grade		0.887	0.593-1.327	0.560			
Stage		1.849	1.355-2.523	1.04E-04	1.849	1.355-2.523	1.04E-04
TCGA set (n =364)							
GILncSig	High/Low	1.091	1.042-1.141	1.68E-04	1.114	1.061-1.168	1.39E-08
Age		1.005	0.991-1.020	0.481			
Gender		0.758	0.513-1.118	0.162			
Grade		1.121	0..865-1.454	0.388			
Stage		1.808	1.463-2.234	4.31E-08	1.866	1.505-2.315	1.11E-05

### Comparison of the GILncSig with existing lncRNA-related signatures in prognosis value

**Figure 5:**
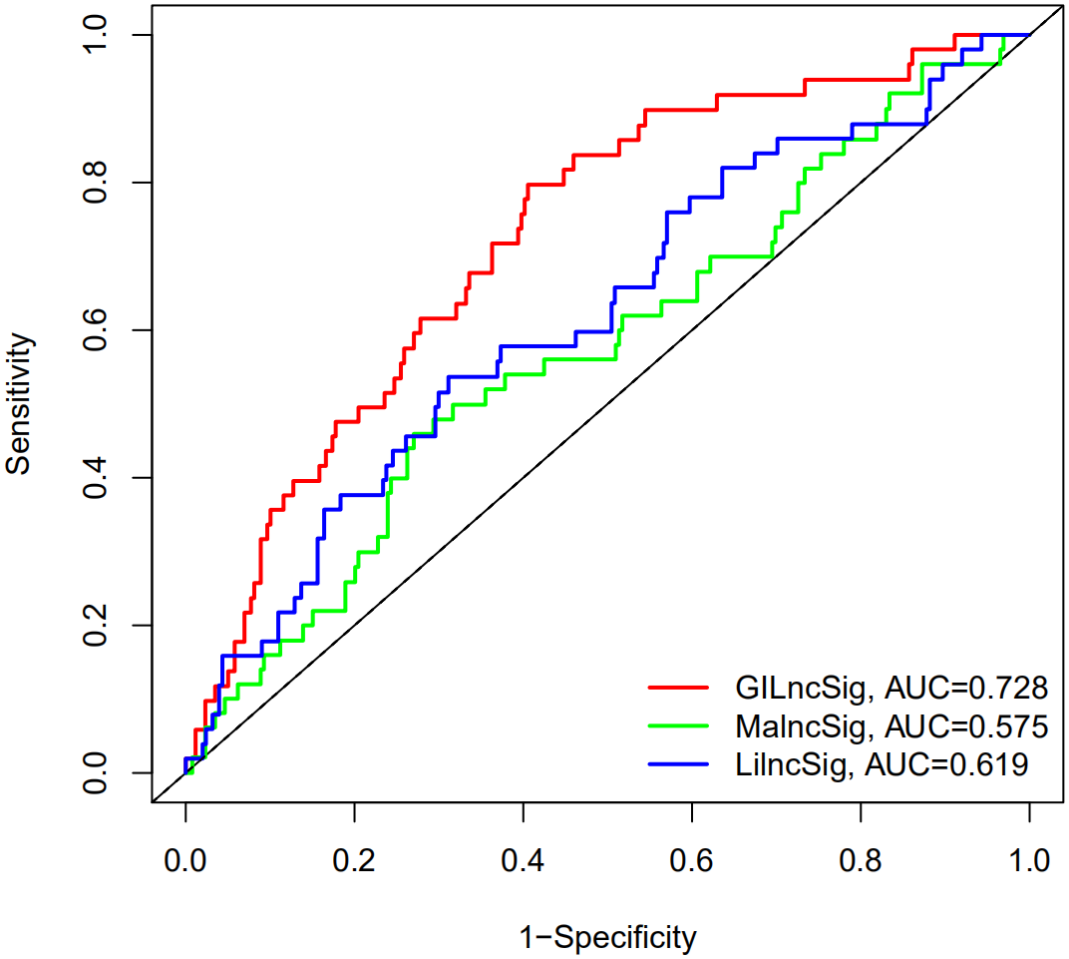
**The ROC analysis of overall survival (OS) for the LilncSig and MalncSig.** The AUC of OS for the GILncSig, LilncSig and MalncSig is 0.728. 0.619 and 0.575, respectively.

To further illustrate the performance of GILncSig in our study, we recruited two recently published lncRNA signatures for survival prediction of HCC patient. Li’s study included 12-lncRNA signature (LilncSig) and Ma’s study included 4-lncRNA signature (MalncSig) [28, 29]. For LilncSig, Li et al. analyzed 12 pairs of HCC and adjacent normal mucosal tissues and identified 3900 differentially expressed lncRNAs as candidate biomarkers for the prognosis of HCC [28]. The 12-lncRNA signature was constructed using the least absolute shrinkage and selection operator (LASSO) cox regression method [8]. For MalncSig, the probe expression profiles of 225 HCC samples and 220 paired non-tumor tissue samples were derived from Gene Expression Omnibus (GEO)-GSE14520 [29]. Univariate coxregression and LASSO model were applied to screen lncRNAs linking to the overall survival. Then the multivariate Cox regression model was implemented to construct the prognostic score model [20]. Comparison analysis was performed between GILncSig and two recruited lncRNA signatures. As shown in Figure 5, the AUC of overall survival (OS) for the GILncSig is 0.728, which is significantly higher than that of LilncSig (AUC = 0.619) and MalncSig (AUC = 0.575). In addition, GILncSig consisted of 3 lncRNAs while MalncSig/LilncSig included 4/12 lncRNA for outcome prediction. Based on the AUC and lncRNA number, our optimized GILncSig significantly outperformed the two recently published lncRNA signatures in the sense of prognostic performance.

### Comparison of the GILncSig with TP53 mutation status in prognosis value.

**Figure 6:**
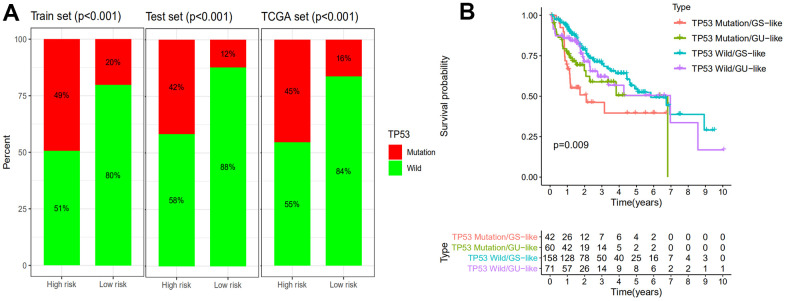
**Comparison of the GILncSig with TP53 mutation status in prognosis value.** (**A**) The proportion of TP53 mutation in high- and low-risk groups in the training set, testing set and the TCGA set. (**B**) Kaplan–Meier curve analysis of overall survival is shown for patients classified according to TP53 mutation status and the GILncSig. Statistical analysis was performed using the log-rank test.

It is reported that mutation of TP53 gene is associated with worse survival in cancer patients [30]. Statistic results suggested that patients in the high-risk group have a significantly higher percentage of TP53 mutations than patients in the low-risk group among the training set, testing set and all TCGA samples (Figure 6A) (*P*<0.001). The proportions of TP53 mutation in the high-risk group of the training set, testing set and TCGA set were 49%, 42% and 45% respectively. In the low-risk group of the training set, testing set and TCGA set, TP53 mutation proportions were 20%, 12% and 16% respectively. TP53 transcriptionally targets hundreds of genes and regulates the expression of gene contributed to cell cycle, apoptosis, DNA repair proteins and metabolic [31, 32]. Recent studies had demonstrated that mutation of TP53 increased genomic instability and served as an independent prognostic marker [33–37]. Therefore, we continued to compare the performance of the GILncSig and TP53 mutation status in prognosis value. According to the GILncSig and TP53 mutation status, we classified all samples into TP53 Mutation/GS−like group, TP53 Mutation/GU−like group, TP53 Wild/GS−like group and TP53 Wild/GU−like group. Figure 6B revealed the survival curve of four risk groups. With regard to GU−like patients, the survival of TP53 Mutation group was more closely resembles that of TP53 Wild group. However, for patients with TP53 mutation, the survival of GU−like group was not similar to the GS−like group. Moreover, the survival outcome of patients in both GU−like group and TP53 mutation group were significantly worse than that in both GS−like group and TP53 wild group, indicating that the GILncSig and TP53 mutation status exhibited better prognostic performance than TP53 mutation status alone.

### Unfavorable impact of AC145343.1 on HCC

**Figure 7:**
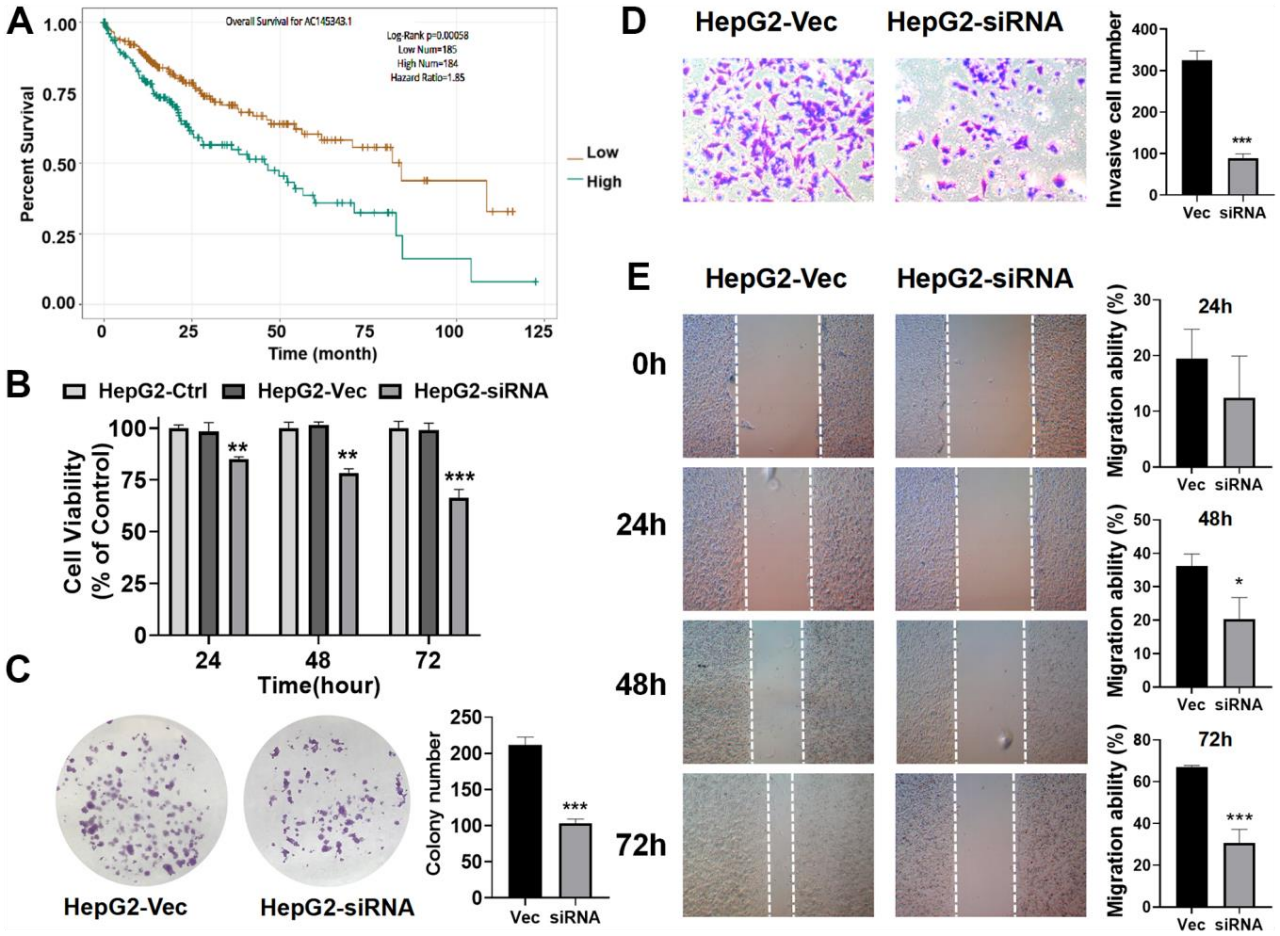
**Unfavorable impact of AC145343.1 on HCC *in vitro*.** (**A**) Kaplan–Meier curve of expression level of AC145343.1 on HCC patients using ENCORI. (**B**) Cell viability of HepG2 was significantly inhibited after AC145343.1 silencing. (**C**) Colony formation number was significantly decreased after AC145343.1 silencing compared to the control group. (**D**) Invasive ability of HepG2 was suppressed after AC145343.1 silencing manifested by transwell experiment. (**E**) Wound healing array demonstrated that AC145343.1-downregulated HepG2 cell exhibited obviously delay in wound healing when compared with the control group. ^*^P<0.05, ^**^P<0.01, ^***^P<0. 01.

Of the GILncSig, AC145343.1 served as a risky factor and the most important lncRNA for prognostic prediction according to the regression coefficient. Hence, we further assessed the function of AC145343.1 with regards to HCC. Firstly, we sought to characterize the potential prognostic ability of AC145343.1 indicated by survival analysis using The Encyclopedia of RNA Interactomes (ENCORI) as described in our previous study [38]. A significant decline of survival time in high AC145343.1 set were observed (Figure 7A). To evaluate the phenotype effect of AC145343.1 *in vitro*, we transfected siRNA into HepG2 cell to establish AC145343.1-downregulated cell line. To assess the proliferation inhibitory potential of AC145343.1 in HCC, we employed CCK-8 and colony formation assay in HepG2 with/without AC145343.1 downregulation. After AC145343.1 silencing, HepG2 exhibited obviously lower cell viability and significantly decreased colony area compared to the control group (Figure 7B, 7C). Invasion and migration roles of AC145343.1 were further observed, manifested by transwell assays and wound healing array. Transwell assay indicated silencing AC145343.1 remarkably decreased the number of HepG2 that migrated across the transwell chamber (Figure 7D). Wound healing array also revealed that AC145343.1-downregulated HepG2 exerted a significant delay in wound healing when compared with the control group (Figure 7E). Taken together, these results supported AC145343.1 knockdown repressed the proliferative, migratory and invasive abilities of HepG2 cells.

## DISCUSSION

The onset is hidden, the early symptoms are not obvious and the clinical manifestations of HCC are quite different making early diagnosis and prognosis difficult. Imaging examination plays an important role in the diagnosis of HCC, but the diagnostic sensitivity is greatly reduced and restricted when the lesion is small [39]. AFP is currently the most widely used biomarker for the diagnosis of HCC with low sensitivity and specificity [13]. Therefore, it is necessary and urgent to look for the new prognostic evaluation indicators in order to improve the prognosis of HCC. With the rapid development of high-throughput sequencing technology, genomic instability-related lncRNA is gradually being discovered to be a potential biomarker of prognostic evaluation indicator [18, 19]. LncRNA plays an important regulatory role in chromosome modification, nuclear transcription and cytoplasmic post-transcriptional processing, and can be used as a tissue factor of subcellular structure to regulate the location or activity of the protein [40, 41]. Abnormal expression of lncRNA is closely related to human diseases, especially in tumors. The abnormal expression of lncRNA has been found in HCC, and it is involved in tumor growth, infiltration, metastasis and recurrence suggesting that lncRNA may become a new prognostic marker in the occurrence and development of HCC [42, 43]. Studies have shown that lncRNA is an emerging regulator of genomic instability, such as BGL3 and NORAD [18, 44]. However, the identification of lncRNAs related to genomic instability and their prognosis and clinical significance for HCC are still unclear. Thus, we constructed the GILncSig with a computational framework integrating somatic mutation information and lncRNA expression profiles to confirm the roles of lncRNAs related to genomic instability in prognosis for HCC.

First, we integrated the lncRNA profile with the somatic mutation profile of HCC for a comprehensive analysis, and obtained 88 lncRNAs with |fold change| > 1.5 and FDR adjusted *P*<0.05 related to genome instability. Then, PCGs closely related to lncRNAs were screened out to perform Go and KEGG pathway analysis. We found that biological processes and biological pathways were mainly involved in various small molecule metabolic/catabolic process, a variety of metabolism pathway, HIF-1 signal pathway, biosynthesis of amino acids and so on. The normal cell cycle is the basic condition to ensure the correct sequence, integrity and fidelity of life activities and study suggests metabolic dysfunction linking to DNA damage causes dysregulated cell cycle, which will lead to genomic instability [45, 46]. In addition, research shows that metalloproteinase SPRTN involved in biosynthesis of amino acids and metabolism regulates covalent DNA-protein crosslinks to prevent genome instability and carcinogenesis [47]. HIF, a major participant in sensing and adapting to hypoxia, is closely related to genome instability and cancer progression [48]. The results of Go and KEGG pathway analysis further proved PCGs closely related to lncRNAs were mainly involved in genome instability, which is an important feature of cancer pathogenesis [46]. We further explored the roles of GILncRNAs in the prognosis prediction of HCC patients and received 3 candidate GILncRNAs including AC145343.1, AC004862.1 and ZFPM2-AS1 by using multivariate cox regression analysis. Patients was divided into two groups according to GILncSig score. The low risk group showed a longer survival time compared with high risk group with significance in the training set. The same result was validated in the testing set and TCGA set by using Kaplan–Meier and time-dependent ROC curve analysis. It is worth noting that the AUC of ROC curve for GILncSig in the training set, testing set and TCGA set were respectively achieved 0.781, 0.665 and 0.728 revealing outstanding performance of GILncSig for prognosis prediction. Meanwhile, multivariate cox regression analysis showed the predictive significance of GILncSig was independent of other clinical factors, further elaborating the reliability of GILncSig for HCC prognosis prediction. Moreover, we found the number of somatic mutation in high risk group was higher than low risk group with significant in testing set and TCGA set, demonstrating GILncSig was significantly associated with HCC mutator phenotype, which is important for assessing genome instability and prognosis. After systematically reviewing the literature, we found that ZFPM2-AS1 upregulated in HCC tissues and involved in cell cycle progression with miR-653 binding sites can reverse the inhibitory effect of miR-653 on the proliferation and metastasis of HCC cells by regulating the target gene GOLM1 of miR-653, and regulate the process of HCC by binding to miR-139 to regulate the expression of GDF10 [49, 50]. However, no previous reports describing the function of AC145343.1 and AC004862.1 until now. We found that the lncRNA AC145343.1 is located in chromosome 17q24 [51]. Genetic variations in the region of chromosome 17q24 are predictors of prostate cancer and lung malignancy risk [52–54]. The AC004862.1 gene is localized in chromosome 7q21.11. Previous studies showed frequent amplification of 7q21 was found in Barrett’ s and gastric cardia cancers and associated with early neoplastic lesions [55, 56]. The amplicon in the 7q21 area is known for breast cancer metastasis-related susceptibility loci in previous genome wide analysis [57]. In this study, we first propose that lncRNA AC145343.1 has great potential to serve as a risk factor and lncRNA AC004862.1 is recognized as a protective factor for HCC prognosis performing crucial role in the development of HCC. However, further research is needed to understand the deeper mechanisms. The present results in our study and available published literatures reveal the GILncSig has great potential to perform prognosis prediction and are very likely to become indicators of genome instability for HCC patients at the same time.

To further confirm the performance in prognosis prediction of GILncSig, we recruited two recently published lncRNA signatures of survival prediction for HCC patient [28, 29]. We found that the AUC for the GILncSig with lower number of lncRNA was higher than that of LilncSig and MalncSig, indicating our GILncSig possessed more optimized prognostic effect. Studies show that TP53 mutation increases genomic instability and serves as an independent prognostic marker [33–37]. Cells with DNA damage can avoid apoptosis then transform into cancer cells in the event of TP53 mutation. In HCC, TP53 alterations are found to be associated with serum AFP level, tumor stage, vascular invasion, tumor differentiation and Child-Pugh class [58–61]. Meanwhile, HCC patients with TP53 mutations have shorter OS and relapse-free survival times [62]. Consistent with previous research, patients in high risk group showed a higher TP53 mutation rate than those in low risk group in training set, testing set and TCGA set, strongly showing GILncSig can reflect the TP53 mutation status. Then, we further compared the prognosis value of GILncSig and TP53 mutation. The survival curve of TP53 Mutation/GU−like group was more closely resembles that of TP53 Wild/GU−like group but not that similar to the TP53 Mutation/GS−like group, indicating that GILncSig rather TP53 mutation was more closely associated with the overall survival of HCC patients, suggesting TP53 mutation status alone does not perform well for predicting outcome of GU−like patients. Remarkably, patients in both GU−like group and TP53 mutation group had a shorter survival time compared to that in both GS−like group and TP53 wild group, indicating that the GILncSig and TP53 mutation status exhibited better prognostic performance than TP53 mutation status alone. Finally, AC145343.1 was regarded as the most relevant one for outcome prediction. A significant decline of survival time in patients with high AC145343.1 was observed, indicating AC145343.1 exerts pro-cancer effect in human HCC among the lncRNAs in GILncSig. Therefore, we conducted the molecular biology experiments *in vitro* to validate the effect of AC145343.1 on HCC. CCK-8 and colony formation assay showed that cell viability and colony area of HepG2 cells with AC145343.1 silencing were significantly reduced, which revealed that AC145343.1 silencing contributed to inhibit the proliferative of HepG2 cells. Further, transwell assays and wound healing array showed that the migration ability of HepG2 cells with AC145343.1 silencing were significantly suppressed, which confirmed that AC145343.1 silencing repressed the migration and invasion of HepG2 cells. All *in vitro* results confirmed that AC145343.1 tended to be a critical risky factor for the survival of HCC patients.

We have provided preliminary evidence for evaluating the relationship between GILncSig and the prognosis of HCC. GILncSig may be of great significance in predicting the degree of genome instability and prognosis of HCC patients. But it still has certain limitations for clinical purposes. Although the prognostic value and independence of lncRNA AC145343.1, ZFPM2-AS1 and lncRNA AC004862.1 on HCC have been verify in the training set, testing set and TCGA set and we proved that AC145343.1 is a high risk factor for HCC *in vitro,* more data sets, *in vivo* experiments, *in vitro* experiments and clinical experiments are still necessary to verify the accuracy, repeatability and the mechanism in regulating genome instability of GILncSig in the future.

## MATERIALS AND METHODS

### Data collection

Clinical characteristics, RNA sequencing (RNA-Seq) data and somatic mutation variation information of HCC patients were obtained from The Cancer Genome Atlas (TCGA) database (https://portal.gdc.cancer.gov/). 377 samples with RNA expression profiles, survival information and common clinical characteristics were extracted for next analysis. Clinical and pathological characteristics of all samples were shown in Supplementary Data 1. Somatic mutation data of 364 HCC patients were also downloaded from TCGA. After integration of 377 samples with their somatic mutation data, 343 patients remained. These patients were randomly separated into two groups according to previous study, named training set and testing set respectively [63]. The training set with 172 HCC patients was used to recognize clinical outcome-related lncRNA signature and establish prognostic risk model. The testing set with 171 HCC patients was used to evaluate the performance of prognostic risk model of training group.

### Genome instability-associated lncRNAs analysis

**Figure 8:**
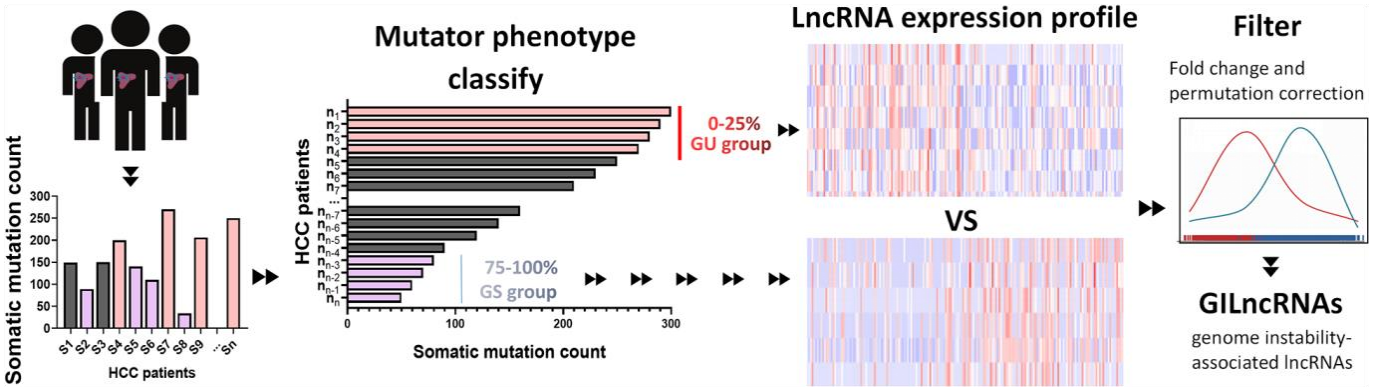
**Computational overview of genomic instability-related lncRNAs.** Somatic mutations of each HCC sample were counted. Samples were divided into two groups, GU group (patients’ mutator phenotype ranked in the top 25%) and GS group (patients’ mutator phenotype ranked in the last 25%). Genomic instability-related lncRNAs were examined according to the difference of lncRNA expression profile between GU group and GS group.

Somatic acquired genomic instability is one of the hallmarks of malignancy cancer [64]. Aberrant lncRNA levels are contributed to abnormal mutation and expression of genes involved in both tumor initiation and progression [65]. To recognize genome instability-associated lncRNAs (GILncRNAs), we constructed a computational frameworks integrating somatic mutator information and lncRNA expression profiles of tumor genome (Figure 8): (i) total cumulative somatic mutations of each sample was calculated; (ii) samples were ranked based on cumulative number of somatic mutations from high to low; (iii) The top 25% samples in the ranking list were set as genomic unstable (GU)-like group while the last 25% were set as genomically stable (GS)-like group; (iv) lncRNA expression profiles between the GU group and GS group were filtered; (v) GILncRNAs were identified according to the criteria: |fold change| > 1.5 and false discovery rate (FDR) adjusted *P*<0.05.

### Statistical analysis

To identify GU group and GS group using profiles of differentially expressed lncRNAs, hierarchical cluster analyses were carried out based on Euclidean distances and Ward’s linkage method. Univariate and multivariate Cox proportional hazard regression analysis was performed to evaluate the prognostic value of the expression level of GILncRNAs. To obtain outcome prediction model using GILncRNAs, we combined the regression coefficients from the multivariate regression analysis with the expression data of identified prognostic lncRNAs and constructed the following equation of genome instability-derived lncRNA signature (GILncSig) according to previous study [63]:


$$\text{GILncSig}\;(\text{sample})\;=\;\sum_{\text{i}=1}^{\text{n}}\text{coef}\;({\text{lncRNA}}_{\text{i}})\;\text{*}\;\text{expr}\;{\text{(lncRNA}}_{\text{i}}\text{)}$$

GILncSig (sample) in the formula is a risk score of the prognosis of HCC patient. lncRNAi indicates the ith prognostic lncRNA and expr (lncRNAi) represents the expression level of lncRNAi of HCC patient. coef (lncRNAi) is the contribution index of lncRNAi to prognostic risk score which was acquired from the regression coefficient of multivariate Cox analysis. Patients were further equally divided into high-risk group and low-risk group according the risk score. High-risk group of unfavorable prognosis with high GILncSig and low-risk group with low GILncSig were obtained to verified the performance of outcome prediction model. To demonstrate the survival rate and median survival of different group, we performed the Kaplan–Meier analysis and *P*<0.05 was considered to be meaningful. Independence validation among GILncSig and other important clinical factors were evaluated using multivariate Cox regression and stratified analysis. Hazard ratio (HR) and 95% confidence interval (CI) were obtained through Cox analysis. R-version 3.5.2 was used to compute ROC curves and ROC AUC to validate the performance of the constructed GILncSig.

### Functional enrichment analysis

To unreal the co-expressed lncRNA-mRNA pairs, Pearson Correlation Coefficients were calculated based on the expression profile between every differentially expressed lncRNA and mRNA. The top 10 items were defined as significant co-expressed mRNAs of lncRNAs. To disclose the biological functional properties associated with the proposed GILncRNAs, functional enrichment analysis of significant co-expressed mRNAs including Gene Ontology (GO) terms and Kyoto Encyclopedia of Genes and Genomes (KEGG) pathway was applied. The enrichment analysis was performed using clusterProfiler software in R-version 3.5.2 [63].

### Cell proliferation and colony formation assays

Human liver cancer cell lines HepG2 were purchased from the Cell Bank of the Type Culture Collection of the Chinese Academy of Sciences, Shanghai Institute of Biochemistry and Cell Biology. Cells were cultured in Dulbecco’ s Modified Eagle Medium (DMEM) supplemented with 10% fetal bovine serum (FBS), 1% penicillin and 1% streptomycin (Gibco Life Technologies, Lofer, Austria), then incubated in a humidified incubator with settled parameters (37° C, 5% CO_2_). AC145343.1 specific siRNA and negative control siRNA were obtained from Vigene Biosciences and transfected into HepG2 using X-tremegene siRNA transfection reagent (Roche Diagnostics, Shanghai, China) according to the standard guidelines.

Cell proliferation ability of HepG2 with/without AC145343.1 downregulation was evaluated by the Cell Counting KIT-8 (CCK-8, KeyGEN BioTECH, Nanjing, China) according to manufacturer's guidelines. Briefly, 3×10^3^ cells suspended were seeded in each well of 96-well plates and incubated overnight for cell attachment. The CCK-8 cell proliferation reagent (10 μl) was added to each well at 24 h, 48 h and 72 h. 4 h after CCK-8 administration, cell proliferation ability is detected. For colony formation assay, 1×10^3^ cells suspended were seeded in each well of 6-well plate and incubated for 2 weeks. Cell colonies were fixed with 4% formaldehyde solution and stained with crystal violet for image visualization.

### Wound healing assay and transwell assay

Migration ability of HepG2 with/without AC145343.1 downregulation was observed by wound healing assay. 5×10^5^ cells suspended were seeded into a 6-well plate. When the cells permeated 90% of the plate, a ‘wound’ in cell monolayers was scratched using a 1 ml pipette tip. Then images of wounds were captured by an inverted microscope at 0 h, 24 h, 48 h and 72 h. Migration ability was calculated by calculating the wound confluence parameter. Invasive ability of cells was further accessed by transwell assay. The transwell chambers were placed in a 24-well plate which contained complete cultured medium (10% FBS), then added a layer of Matrigel before cell seeding. 8×10^4^ cells were seeded into upper transwell chambers and inserted into 300 μl serum-free medium. The cells of the upper surface were removed using a cotton swab 24 h later. Then the cells on the bottom surface were fixed with 4% formaldehyde solution and stained with 0.5% hematoxylin solution for 20 min. Images of invaded cells were collected by an inverted microscope.

### Availability of data and materials

The datasets used during the current study are available from TCGA database (https://cancergenome.nih.gov/), and Supplementary Materials.

## Supplementary Material

Supplementary Tables

Supplementary Data 1

Supplementary Data 2
