# Supplementary material for: Construction of a genome instability-derived lncRNA-based risk scoring system for the prognosis of hepatocellular carcinoma
Source: Aging (Albany NY). 2021 Nov 18;13(22):24621–39. doi: 10.18632/aging.203698 (PMC8660619; doi:10.18632/aging.203698)
Supplement: Supplementary Tables [file aging-13-203698-s001.pdf]

SUPPLEMENTARY TABLES

Supplementary Table 1. The common clinical features characteristic between the training set and testing set.

Variables	Type	Total	Test	Train	Pvalue
Age	<=65	216(62.97%)	107(62.57%)	109(63.37%)	0.967
	>65	127(37.03%)	64(37.43%)	63(36.63%)	
Gender	FEMALE	110(32.07%)	63(36.84%)	47(27.33%)	0.0763
	MALE	233(67.93%)	108(63.16%)	125(72.67%)	
Grade	G1-2	214(62.39%)	106(61.99%)	108(62.79%)	1
	G3-4	124(36.15%)	61(35.67%)	63(36.63%)	
Stage	Stage I-II	238(69.39%)	115(67.25%)	123(71.51%)	0.9667
	Stage III-IV	83(24.2%)	41(23.98%)	42(24.42%)	
T	T1-2	252(73.47%)	126(73.68%)	126(73.26%)	0.9524
	T3-4	88(25.66%)	43(25.15%)	45(26.16%)	
M	M0	245(71.43%)	113(66.08%)	132(76.74%)	0.8991
	M1	3(0.87%)	2(1.17%)	1(0.58%)	
N	N0	239(69.68%)	111(64.91%)	128(74.42%)	0.9081
	N1-3	3(0.87%)	2(1.17%)	1(0.58%)	

Supplementary Table 2. Univariable Cox regression analysis.

Gene symbol	HR	95% CI	P-value
CASC9	1.068	1.012-1.126	0.017
MIR210HG	1.157	1.062-1.261	9.90E-04
LUCAT1	1.174	1.078-1.278	2.33E-04
PRRT3-AS1	1.091	1.028-1.156	0.037
KCNMB2-AS1	1.235	1.082-1.409	1.72E-03
AC145343.1	1.378	1.072-1.772	0.012
AL731684.1	1.141	1.037-1.255	0.007
AC004862.1	0.811	0.683-0.964	0.017
ZFPM2-AS1	1.148	1.095-1.204	1.14E-08
